# Influence of Vanadium Microalloying on Microstructure and Property of Laser-Cladded Martensitic Stainless Steel Coating

**DOI:** 10.3390/ma13040826

**Published:** 2020-02-12

**Authors:** Wenfeng Hu, Hongmei Zhu, Jipeng Hu, Baichun Li, Changjun Qiu

**Affiliations:** Provincial Key Laboratory of Advanced Laser Manufacturing Technology, University of South China, Hengyang 421001, China; huwenfeng106@126.com (W.H.); hujipeng106@126.com (J.H.); libaichun106@126.com (B.L.)

**Keywords:** laser cladding, martensitic stainless steel, vanadium microalloying, microstructure, mechanical properties

## Abstract

Martensitic stainless steel (MSS) coatings with different vanadium (V) contents (0–1.0 wt%) by microalloying have been successfully fabricated utilizing a unique laser cladding technique. The microstructure and properties of the resulting MSS coatings, with and without element V addition, have been carefully investigated by various advanced techniques, including XRD, SEM, TEM, microhardness tester, universal material testing machine, and electrochemical workstation. It was found that the V-free coating was mainly composed of martensite (M) and ferrite (F), trace *M*_23_C_6_ and *M*_2_N, while the V-bearing coatings consisted of M, F, *M*_23_C_6_, and VN nano-precipitates, and their number density increased with the increase of V content. The V microalloying can produce a significant impact on the mechanical properties of the resulting MSS laser-cladded specimens. As the V content increased, the elongation of the specimen increased, while the tensile strength and microhardness increased firstly and then decreased. Specifically, the striking comprehensive performance can be optimized by microalloying 0.5 wt% V in the MSS coating, with microhardness, tensile strength, yield strength, and elongation of 500.1 HV, 1756 MPa, 1375 MPa, and 11.9%, respectively. However, the corrosion resistance of the specimens decreased successively with increasing V content. The microstructure mechanisms accounting for the property changes have been discussed in detail.

## 1. Introduction

Over the past several decades, the laser cladding technique has received much attention in academic research and industrial applications because of its inherent merits, such as metallurgical bonding, low dilution ratio, small heat-affected zone, accurate process control, and high efficiency [1,2]. It is well-known that Fe-based laser cladding coatings have exhibited many advantages including its low cost, closing to the composition of the substrate and acceptable compatibility. Among them, martensitic stainless steel (MSS) has been considered as one of the most potential materials owing to its high strength, moderate corrosion resistance and tailored properties by the subsequent high-temperature treatment.

Up to date, there were only a few studies in the literature to report microalloying of the laser-cladded MSS coatings, e.g., elements of molybdenum (Mo) [3,4,5,6], niobium (Nb), and boron (B) [4,6], as well as additives of vanadium carbide (VC) [7] or titanium carbide (TiC) [8]. The aforementioned elemental additions have demonstrated to improve wear resistance [4], mechanical properties [7,8], and also corrosion resistance [3,5,7] of the laser-cladded MSS coatings. However, the higher melting points, such as 2623 °C for the refractory element Mo [3] and >3000 °C for ceramic additive TiC [8], necessitate higher laser energy input, which may cause the loss of alloying elements during the high-temperature processing. Microsegregation and microcracks were prone to generate in the B-alloying laser clads [4]. In addition, the poor wettability and large heat expansion discrepancy between the ceramic additives and the MSS matrix led to a low plasticity and toughness [8].

As is well known, the addition of vanadium (V) has produced a significant impact on the microstructure and property of the traditionally manufactured Fe-based alloys through two ways. (i) Grain refinement—Medina et al. [9] reported a nearly 50% reduction in the grain size due to the intragranular nucleation of ferrite on vanadium nitride (VN) precipitates in a hot strained 0.15% V-microalloyed steel. Yang et al. [10] found that the nano-sized V-enriched carbide particles formed during reheating a high-strength and low-alloy martensite steel, exerting a pinning effect to the grain growth of austenite with an average size of 3.5 µm. (ii) Precipitation strengthening—Gwon et al. [11] revealed a significant increase of yield strength resulting from micro-alloying vanadium (0–0.3 wt%) in a TWIP steel, which was estimated to be 413 MPa/V wt%. The VC nano-precipitates were found to produce an effective precipitation hardening effect. Li et al. [12] studied the precipitation behavior and microstructural evolution of vanadium-added martensitic steel, and also found that the precipitation strengthening contribution of the nano-sized VC particles was 167 MPa by the Ashby-Orowan mechanism.

To our best knowledge, there were little reported on the addition of element V in the laser-cladded MSS coatings. This study aims to fabricate the MSS coatings with different V contents by the unique and effective laser cladding technique, and investigate the consequent influence of V microalloying on the microstructure and properties of the as-achieved MSS coatings in detail. This work will be greatly beneficial for the laser forming high-strength steel coatings/components with excellent mechanical properties and exemplary corrosion resistance.

## 2. Materials and Methods 

A Q235 steel plate with dimensions of 100 × 60 × 15 mm was used as a substrate, and the surface was sequentially ground with SiC paper of grit scale 200–2000 and cleaned with acetone prior to the laser cladding process. Gas-atomized MSS powders containing different V contents, with a mean particle diameter of 75 µm, were deposited on the substrate as the MSS coatings by the laser cladding technique. The nominal chemical compositions of the substrate and powders, and the corresponding specimen denominations are listed in Table 1.

**Figure 1:**
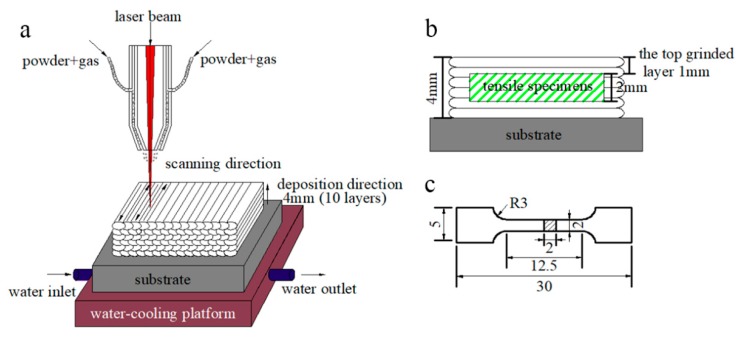
(**a**) Laser cladding process; (**b**) schematic of the sectioned tensile specimen from the coating; and (**c**) geometry of the tensile specimen in mm scale.

A 1.5-kW fiber laser with a coaxial powder feeding and water cooling system was used to fabricate the multi-layer laser cladding, as sketched in Figure 1a. The key processing parameters were modified and adjusted as followings: laser power density of 430 W·mm^−2^, laser scanning speed of 8 mm·s^−1^, overlapping rate of 50% and powder delivery rate of 4 g·min^−1^. High purity nitrogen (99.999%) was used as both a shielding gas and a carrier gas with a flow rate of 10 L·min^−1^. After 10 cladding layers were deposited, the MSS coatings with a dimension of 90 × 40 × 4 mm were fabricated successfully.

To achieve a flat surface, the excessive top ~1 mm in thickness of the laser-cladded MSS coatings were firstly machined off using a grinding machine (Figure 1b). Then, the experimental specimens were extracted from the coatings and mechanically polished, and the geometry of the tensile specimens is shown in Figure 1c. The phase constitution was carefully characterized by a Miniflex600 X-ray diffractometer (XRD), with a Cu-K_α_ radiation (wavelength: 1.5406 Å) at 40 kV and 40 mA. Specimens were scanned in the 2θ range from 20° to 90° with a step size of 0.02° per step. The microstructure of the laser-cladded coatings was investigated by a MERLIN compact scanning electron microscope (SEM, Carl Zeiss AG, Oberkochen, Germany) with energy dispersive spectrometer (EDS). The working distance and accelerating voltage of SEM were about 10 mm and 20 kV, respectively. The in-depth insights on the microstructural features of the coatings were performed by a JEM-2100 transmission electron microscope (TEM, JEOL Ltd., Tokyo, Japan) operated at the acceleration voltage of 200 kV.

Furthermore, the microhardness of the as-achieved specimen was measured by a HVS-1000 micro-hardness tester (Shanghai Precision Instrument Co., Ltd., Shanghai, China) with a load of 200 g and a dwell time of 10 s. The room-temperature tensile properties were determined by a PWS-E100 universal testing machine (Jinan Times Shijin Testing Machine Co., Ltd.,Jinan, China) with a cross-head speed of 0.2 mm·min^−1^. The specimens used for electrochemical tests were polished and mounted in epoxy resin, leaving an exposed area about 1 cm^2^ in contact with 3.5 wt% NaCl solution. Electrochemical measurements were performed at room temperature using the CS300 electrochemical workstation with a conventional three-electrode cell system (Wuhan Questt Asia Technology Co., Ltd., Wuhan, China). The reference electrode was a saturated calomel electrode (SCE), while the Pt electrode and the specimen were used as the counter electrode and working electrode, respectively. The potentiodynamic polarization curves were obtained with a potential range from −1.5 V to 1.0 V at a scan rate of 10 mV/s.

## 3. Results and Discussion

### 3.1. Phase Analysis

**Figure 2:**
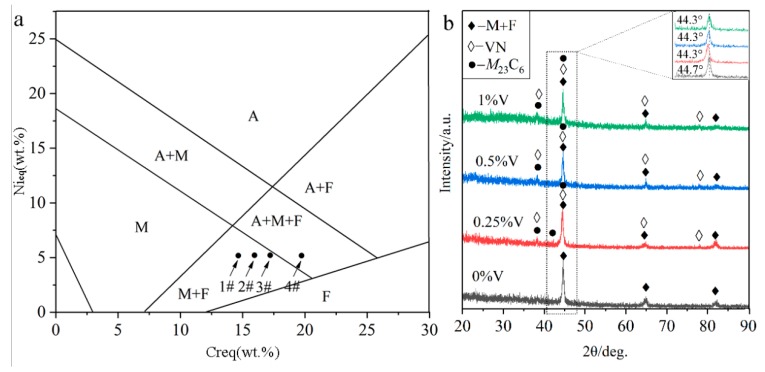
Phase constituent of the laser-cladded MSS coatings with different V contents: (**a**) Schaeffler phase diagram; and (**b**) XRD pattern.

It is generally accepted that the solidification mode of steels under the traditional cooling condition can be inferred by the Schaeffler phase diagram [13], as shown in Figure 2a. The abscissa Cr_eq_ (chromium equivalent) and the ordinate Ni_eq_ (nickel equivalent) are determined by the Schaeffler equivalent formulas (1) and (2) as [13]

Cr_eq_ = Cr + Mo + 1.5Si + 5V (1)

Ni_eq_ = Ni + 30C + 0.5Mn (2)

where each chemical element symbol represents the corresponding weight percentage content (wt%). By calculating the formulas (1) and (2), the Ni_eq_ value is 4.27 and the Cr_eq_ values for specimens 1#, 2#, 3#, and 4# are 14.69, 15.94, 17.19, and 19.69, respectively. As seen from Figure 2a, the higher Cr_eq_ value, the more primary phases ferrite (F) and austenite (A). The solidification process could be described as followings: Once the primary phase δ ferrite was formed, the Cr and other F stabilizers were continuously consumed. Meanwhile, Ni, C, and other austenite (A) stabilizers may be gathered in the inter-dendritic region of the primary F, and subsequently, the peritectic-eutectic reaction occurred to form A. Finally, the A replaced almost the primary F and further continued to transform into martensite (M). Therefore, Figure 2a shows the F, M, and A contents under traditional solidification conditions in the Schaeffler diagram. It can be inferred from Figure 2a that, the specimens 1#, 2#, and 3# are mainly consisted of M and F, while a portion of A still remains in the specimen 4#. However, the cooling rate of the laser cladding technique reaches 10^4^ to 10^6^ °C/s, much higher than the conventional cooling rate of 10^3^ °C/s [13]. Therefore, the formation of a higher proportion of M can be expected under our reported conditions.

The XRD analysis (Figure 2b) indicates that the V-free MSS coating (1#) is mainly composed of M + F. In contrast, a few newly emerged diffraction peaks corresponding to carbide (*M*_23_C_6_, *M* represents Cr, Fe, etc.) and nitride (VN) were detected in the V-bearing MSS coatings (2#~4#), suggesting that the V addition promoted the precipitation of carbide *M*_23_C_6_ and VN. It is worth mentioning that there are not any peaks for austenite detected in the specimen 4# with 1% V content, as inferred in Figure 2a, possibly due to the low content beyond the detection limit of XRD instrument. Interestingly, the (110) diffraction peaks for M in all the V-bearing specimens shifted to a lower angle region (44.3°), in comparison to that (44.7°) in the V-free specimen as shown in the inset of Figure 2b. According to the Brag’s diffraction law, it can be probably ascribed to the lattice distortion, which was caused by the solid solution of the microalloying element V with a larger atomic radius (134 pm) than the matrix Fe (126 pm) [14]. This is similar to the findings reported by Wang et al. [15] that the addition of Al with a larger atomic radius than any other atoms (except Ti) resulted in the left shifting of the XRD diffraction peaks for a high-entropy alloy. Noticeably, the martensite peaks remain in the same positions when the V content exceeds 0.25%. This can be attributed to the extremely limited solubility of V in martensite at room temperature [16], and the increasing V content (0.5%, 1%) will lead to a partial solid solution of V atoms and also the precipitation of V-enriched particles as described hereinafter.

### 3.2. Microstructure Characterization

**Figure 3:**
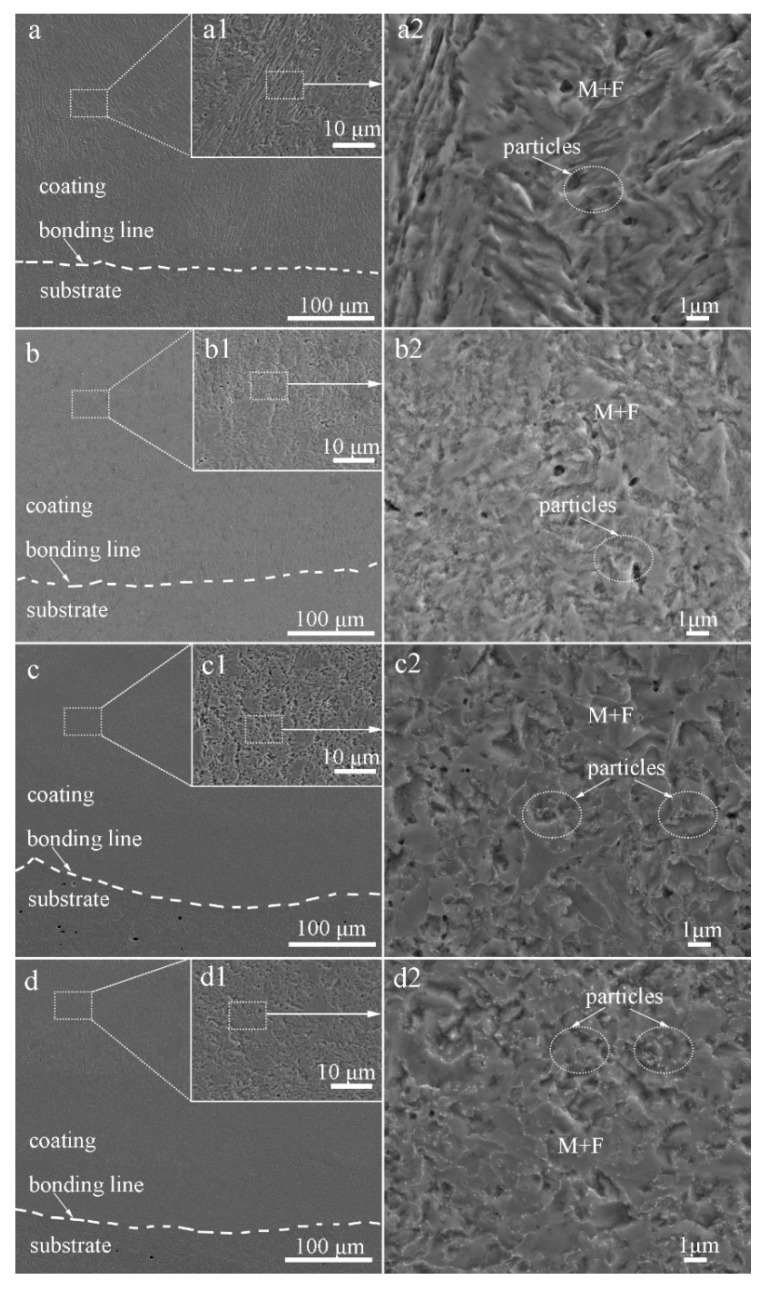
SEM images of the laser-cladded MSS coatings with different V contents: (**a**) 1# (0 wt%V); (**b**) 2# (0.25 wt%V); (**c**) 3# (0.5 wt%V); and (**d**) 4# (1 wt%V).

Figure 3 shows SEM micrographs of the cross-section of the laser-cladded MSS coatings with different V contents. It can be clearly seen that all the laser-cladded MSS coatings were free of defects, such as pores and cracks, and exhibited a superior metallurgical bonding with the substrate. By comparing the specimens demonstrated in Figure 3a1–d1, the coatings were found to compose mainly of lath-shaped martensite, and refined with increasing V content gradually. This is consistent with the results reported by Hui et al. [17] that the microstructure of a medium carbon steel became finer and more uniform with the increasing content of the microalloying element V (0.15~0.45%). The grain refinement effects are generally associated with two aspects: (i) the precipitation pining effect generated by the fine vanadium carbide VC and/or vanadium nitride VN particles; and (ii) the solute dragging effect caused by suppressing the grain boundary migration through the solute dissolved in the austenite [9,10,11]. The existence of the fine V-bearing particles, confirmed by the XRD and SEM results, is mainly responsible for the refined grain size of the laser-cladded coatings.

As encircled in Figure 3a2–d2, the granular particles were observed to distribute over the M matrix homogeneously, and the amounts increased noticeably with V contents increasing. The volume fractions of these particles in specimens 1#, 2#, 3#, and 4#, calculated by Software ImageJ, are 1.36%, 5.23%, 12.14%, and 13.56%, respectively. It was reported that the element V could enhance the driving force for the nucleation of carbide *M*_23_C_6_ and VN [7,18,19], leading to a high number density of small discrete particles distributed over the M matrix, as seen in Figure 3c2,d2. Meanwhile, the precipitation of the carbides depleted the surrounding region of carbon and impeded the diffusion of carbon atoms, consequently promoted the formation of ferrite [19,20]. This is in good agreement with that more ferrite formed with increasing V contents, as schematically shown in Figure 2a.

**Figure 4:**
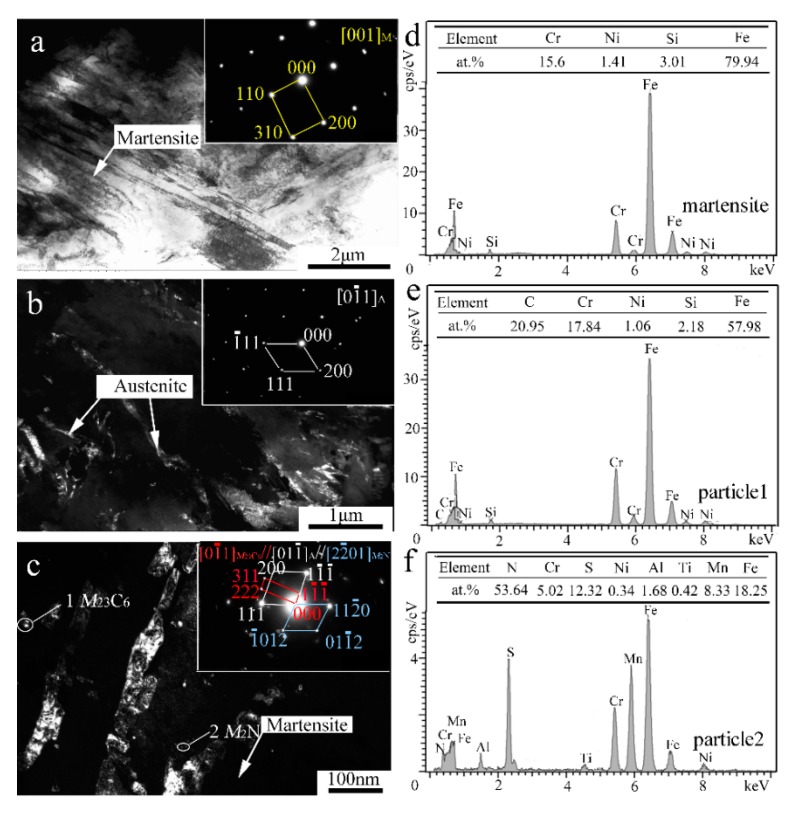
TEM results of the V-free MSS coating: (**a**) Bright-field (BF) image and SAED pattern of M; (**b**) Dark-field (DF) image and SAED pattern of A; (**c**) DF image and SAED pattern of *M*_23_C_6_; (**d**) EDS analysis of martensite; and (**e**,**f**) EDS analysis of particles 1 and 2 denoted in (**c**), respectively.

**Figure 5:**
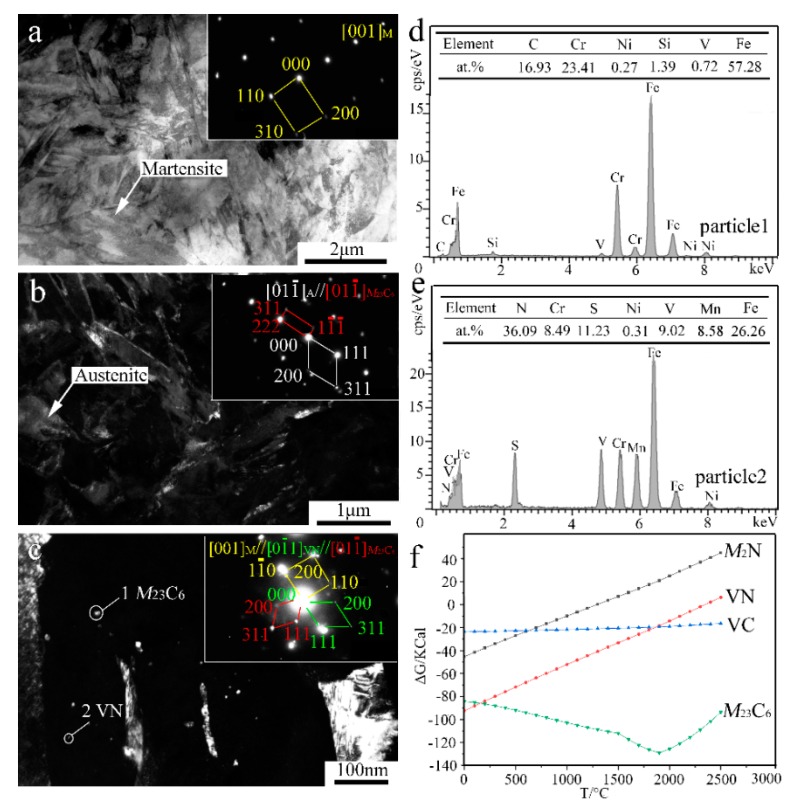
TEM results and Gibbs free energy (ΔG) analysis of the 0.5% V-bearing MSS coating: (**a**) BF image and SAED of M; (**b**) DF image and SAED of A and *M*_23_C_6_; (**c**) DF image and SAED of *M*_23_C_6_ and VN; (**d**,**e**) EDS analysis of particles 1 and 2 denoted in (**c**), respectively; and (**f**) variation relationship between ΔG and temperature.

In order to reveal the in-depth microstructure details, TEM characterization of the V-free and 0.5% V-bearing MSS coatings were carried out as seen in Figure 4 and Figure 5, respectively. There are three distinct particles in the V-free MSS coating, including lath M with width of ~200 nm (Figure 4a), irregular A with width of 100~400 nm (Figure 4b) and granular ones with diameter of 5~10 nm (Figure 4c). The indexed selected-area electronic diffraction (SAED) pattern, as indicated in the inset of Figure 4c, demonstrates the co-existence of *M*_23_C_6_ carbide and *M*_2_N nitride, consistent well with our previous work [21]. The formation of *M*_2_N nitride was promoted because of the in-situ laser nitriding effect during the laser cladding process under the N_2_ atmosphere. The element N could promote the nitride precipitation while suppress coarse eutectic carbide simultaneously, owing to the higher A stability upon quenching and the higher binding energy of Cr-N than that of Cr-C [21]. Interestingly, these two granular particles can be easily distinguished by the EDS analysis as shown in Figure 4e,f. The bigger ones (~5 nm) and smaller ones (~2 nm) could be identified as *M*_23_C_6_ carbide and *M*_2_N nitride, respectively. It is worth mentioning that the diffraction patterns of the M and F were mostly overlapped due to the same crystal structure and similar lattice parameters [22]. Therefore, M and F are hardly distinguished in TEM diffraction patterns simultaneously.

In contrast, the lath-shaped M was obviously refined after a 0.5%V addition in the laser-cladding MSS coating, as seen in Figure 5a. The addition of V element can promote the formation of VN [23], and the intragranular nucleation of maretnsite/ferrite on VN precipitates leads to a remarkable decrease in the grain size [9,10]. Similar as shown in Figure 4c, there are two granular particles with different sizes present in the V-bearing coating in Figure 5c. The SAED index and EDS analysis spectrum of the particles are shown in Figure 5c–e, indicating that the bigger ones (~5 nm) remain *M*_23_C_6,_ while the smaller ones (~1 nm) are VN instead of *M*_2_N in V-bearing MSS coating.

In order to elucidate the mechanisms regarding the formation of the V-bearing particles in the form of VN, as evidenced in the inset of Figure 5c,e, the Gibbs free energies (ΔG) of Cr_23_C_6_, *M*_2_N, VN, VC, as a function of temperature were calculated using HSC Chemistry v7.0 software (Figure 5f). Given that the temperature of the laser molten pool was lower than 1900 °C [24], the order of the calculated values can be described as follows: ΔG_Cr23C6_ < ΔG_VN_ < ΔG_VC_ < ΔG*_M_*_2N_. This suggests that the preferential precipitation is Cr_23_C_6_, VN, VC, and *M*_2_N in sequence. It is worth noting that the chemical bonding between the atom V and the neighbor atom C/N shows both covalent and ionic characteristics. An electron exchange may take place between the atom V and the atom C/N along the V-C/N direction, however, the N atom can obtain more electrons from V in comparison to the C atom [23]. In fact, the iconicity of V-N is stronger than that of V-C due to the larger charge of N atom than C atom. Therefore, the element V is more chemically reactive with interstitial N to form VN in the V-bearing MSS coatings.

**Figure 6:**
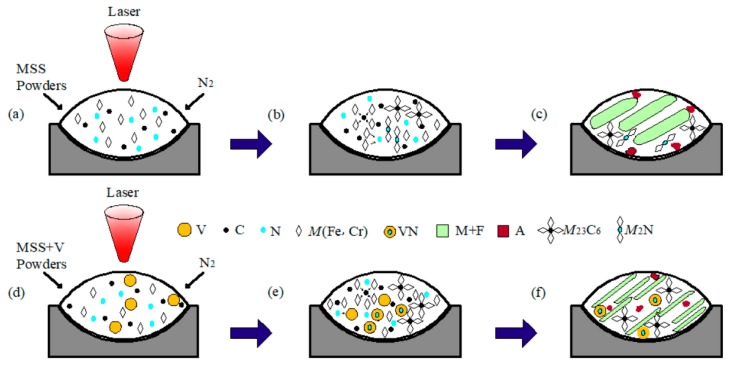
Schematic illustration showing the formation mechanism of the composite particles fabricated by laser cladding: (**a**) MSS powders; (**b**) formation of *M*_23_C_6_ and *M*_2_N particles; (**c**) microstructure in the V-free coating; (**d**) V-bearing NSS powders; (**e**) formation of *M*_23_C_6_ and VN particles; and (**f**) microstructure in the V-bearing coating.

Figure 6 schematically depicts the possible formation mechanism of the composite particles during laser cladding the V-free and V-bearing MSS coatings. When laser beams irradiated on the surface of the preset cladding layer under the N_2_ protective atmosphere, the preset layer was heated rapidly and in situ nitrided by the high energy absorbed (Figure 6a,d). The Marangoni convection facilitated the recombination of Fe, Cr, V, N, and C atoms in the molten pool, and consequently to form *M*_23_C_6_ and *M*_2_N in the V-free MSS coating (Figure 6b,c), *M*_23_C_6_ and VN in the V-bearing MSS coating (Figure 6e,f) based on Figure 4 and Figure 5, respectively.

### 3.3. Mechanical Properties

**Figure 7:**
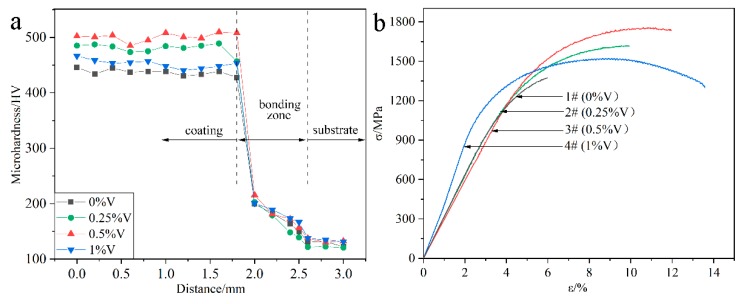
Mechanical properties of the laser-cladded coatings with different V contents: (**a**) microhardness distribution; and (**b**) tensile curves.

Figure 7a presents the microhardness distribution of the laser-cladded specimens with and without V addition, by performing the measurements across the coating, bonding zone and substrate, respectively. In comparison to the average microhardness of the V-free MSS coating (1#, 439.6 HV), the microhardness of the V-alloyed MSS coatings increased firstly and then decreased with the V content increasing. The specimen 3# with a 0.5% V addition exhibits the highest microhardness of 500.1 HV, with ~14% increment than that of the V-free specimen 1#. Noticeably, the coating microhardness reveals a negligible fluctuation with small errors, indicating that a homogeneous microstructure was formed (Figure 3).

Figure 7b shows the tensile curves of the laser-cladded coatings with and without V addition, and the corresponding tensile properties are listed in Table 2. Similar to the microhardness variation (Figure 7a), the tensile strength of the laser-cladded coatings increases firstly and then decreases with increasing V content. Noticeably, the specimen 3# with a 0.5% V addition exhibits the optimal mechanical properties, with ultimate tensile strength (R_m_) of 1756 MPa, yield strength (R_e_) of 1375 MPa and elongation of 11.9%, respectively.

The reasons accounting for the microhardness and tensile strength change of the specimens could be associated with the following factors: On the one hand, the formation of the refined martensite, nano-sized precipitates VN and Cr_23_C_6_ can generate fine-grain strengthening and second-phase strengthening effects, which significantly enhances the microhardness and tensile strength [3,8,25]. On the other hand, the ferrite stabilizer element V can promote the F phase formation [17]. The microhardness of M and F reaches 525.4 HV [26] and 268 HV [27], respectively. Therefore, the increasing portion of F accounts for the reduction in the microhardness and strength, but the enhancement in the toughness of the specimens with higher V addition, consistent with the previous study [28].

Table 2 lists the data of the R_m_, R_e_, and elongation of the laser-cladded MSS coatings with and without V addition developed in this study, and is compared with other Fe-based MSS coatings prepared by the laser cladding technique from the literature [8,21,25,29,30,31]. It can be apparently concluded that the 0.5%V-bearing MSS coating achieved in this work exhibits a striking combination of high strength and high ductility.

**Figure 8:**
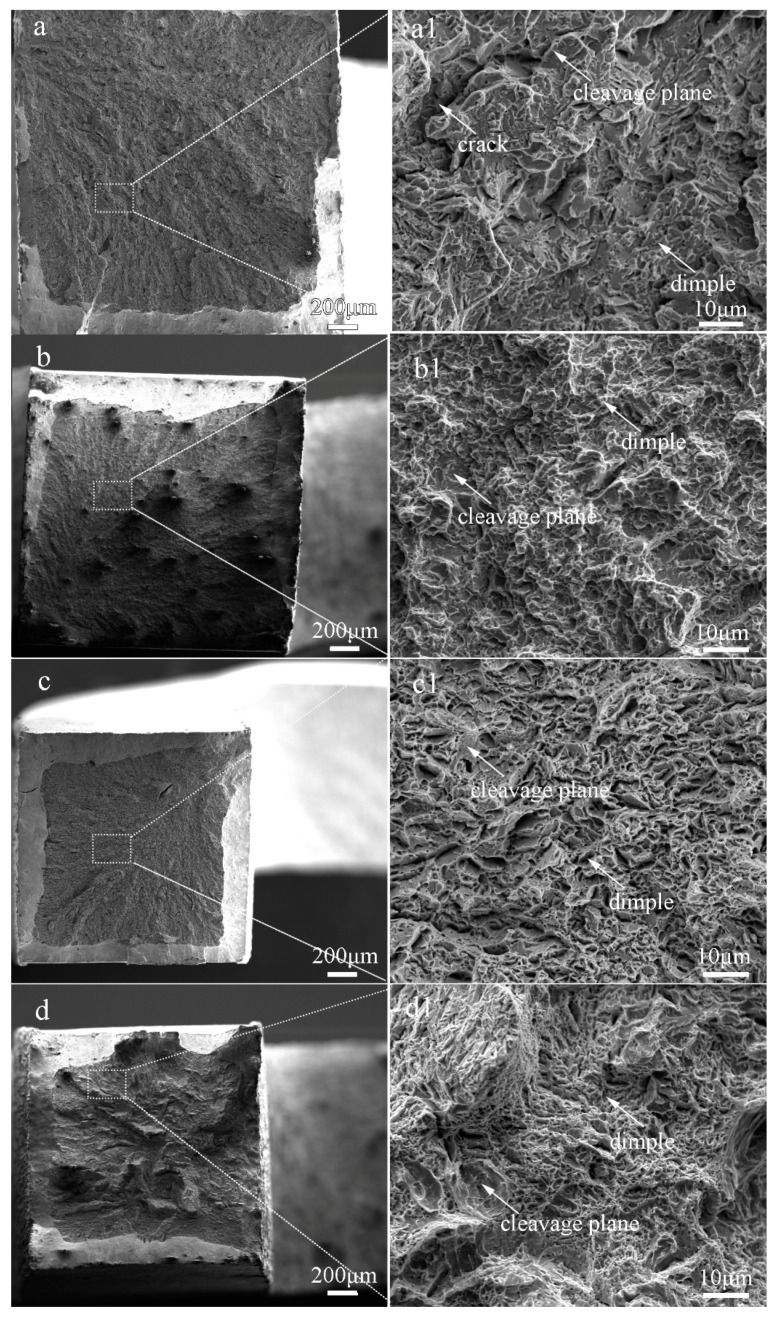
Fracture morphologies of the laser-cladded coatings with different V contents. (**a**) 1# (0 %V); (**b**) 2# (0.25 %V); (**c**) 3# (0.5 %V); and (**d**) 4# (1 %V).

The fractographs of the laser-cladded coatings with and without V addition are shown in Figure 8. Apparently, the fracture surface of the V-free specimen 1# was consisted of massive tearing edges, cracks and large cleavage facets (Figure 8a1), showing a typical characteristic of brittle fracture. In comparison, shallow dimples and small cleavage facets were present in the 0.25%V-bearing specimen 2# (Figure 8b1). Moreover, massive dimples and a few small cleavage planes can be discernible in specimens 3# and 4# with higher V contents (Figure 8c1,d1), consistent well with the tensile result that the ductility of the V-bearing specimen enhanced with increasing V content (Table 2). 

The variation of the tensile behavior with the V content may be attributed to the following aspects: (i) The V-free specimen 1# is composed of coarse lath-shaped martensite, as seen in Figure 3a2. The tensile strain in the martensite region was increased during tensile deformation, leading to an improvement in the strain hardenability. Besides, the joint part of the martensite grains acted as a preferential formation site for microcracks. The number of the microcracks rapidly increased with increasing tensile strain, causing the lower necking deformability and lower ductility [32]. (ii) In contrast, the V-bearing specimens are consisted of fine lath-shaped martensite and nano-sized precipitates (Figure 3 and Figure 5), and the volume fraction of F and A increases with increasing V content. Due to the significant difference in hardness between the M matrix and the neighboring F/A constituent, the hard matrix subjected to the tensile load usually undergoes an initial plastic deformation, leading to a considerable local stress concentration around the soft F and A constituents. Under the combined contributions of fine M, soft F and A, together with the nano-sized precipitates VN and *M*_23_C_6_ particles, more micro-voids were formed triggering a ductile fracture in the V-bearing specimens. A refined M together with higher volume fraction of soft F and A can account for the increased ductility with increasing V content, similar to the effects of V microalloying in a traditional steel [17,28].

### 3.4. Electrochemical Properties

**Figure 9:**
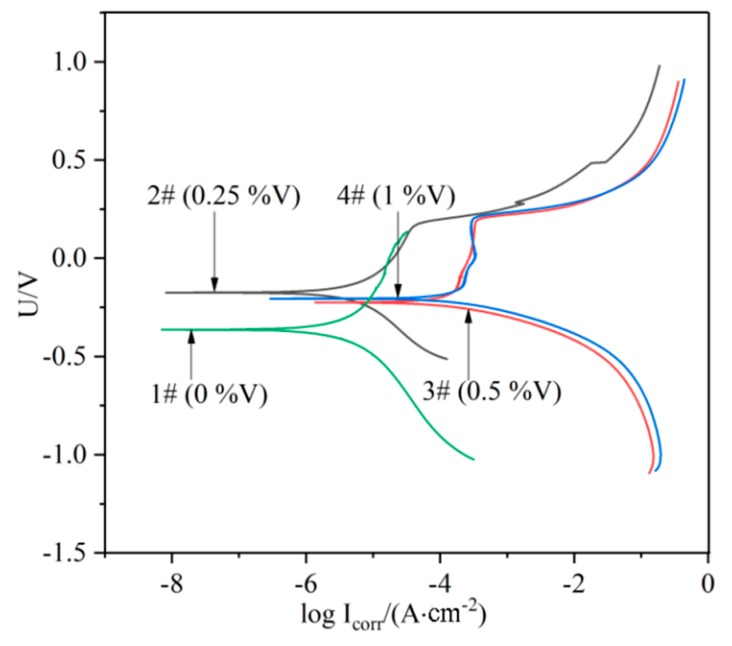
Polarization curves of the laser cladding layers with different V contents.

The potentiodynamic polarization curves for the laser-cladded coatings with and without V addition in a 3.5% NaCl solution are shown in Figure 9. The corrosion potential (*E*_corr_) and corrosion current (*I*_corr_) were measured using Tafel analysis, and the values were given in Table 3. The relatively low value of *I*_corr_ (7.0021 × 10^−6^ A·cm^−2^) for the V-free specimens indicates a lower corrosion rate, while the 0.25%V specimen exhibits the lowest *I*_corr_ (3.4652 × 10^−6^ A·cm^−2^) and the most positive *E*_corr_ (−0.17458 V), implying that the specimen 2# (0.25%V) possesses the best corrosion resistance. Apparently, the polarization curves of the V-bearing coating shifted to the rightward (higher current density) and downward (less noble corrosion potential) for the specimens 3# and 4# with higher V contents (0.5% and 1%). Therefore, it can be concluded that the corrosion resistance of laser-cladded coatings increased firstly and then decreased with the increase of V content, successively.

The possible reasons accounting for the corrosion resistance change could be associated with the following factors. (i) The elements Cr would form passivation film during the laser cladding process, and protect the specimen in the corrosion solution [33]. Therefore, the corrosion resistance of V-free specimen is relatively high. (ii) The addition of 0.25% V element led to the precipitation of a small amount of VN instead of Cr-enriched *M*_2_N in the V-free coating, and thus the corrosion resistance was improved. However, the corrosion resistance began to decrease due to a higher precipitation of nitride and carbide in the specimens with higher V addition as shown in Figure 9, which could preferentially act as the initiation sites for pitting corrosion [34]. (iii) The precipitation of Cr-rich *M*_23_C_6_ carbides consumed a portion of element Cr. This may result in the occurrence of Cr-depleted zones in vicinity, which weaken the formation of passive Cr_2_O_3_ film preventing the Fe-based MSS coating from the corrosion [33,35]. Therefore, the promoted precipitation of *M*_23_C_6_ carbides by V microalloying can be partially responsible for the deterioration of the V-bearing specimen with higher V contents (0.5–1.0%).

## 4. Conclusions

The martensitic stainless steel coatings with different V contents have been successfully fabricated on the surface of a Q235 steel substrate by the laser cladding technique in this work. A few interesting results have been obtained, as demonstrated below:

(1) Unique phase constitution. The V-free MSS laser-cladded coating was mainly composed of M, F, and trace *M*_23_C_6_ and *M*_2_N, while *M*_23_C_6_ and VN are promoted in the V-bearing MSS laser-cladded coatings.
(2) Excellent mechanical properties. The element V could improve the mechanical properties of the MSS laser-cladded coatings by the combination of grain refining strengthening and precipitation strengthening. The microhardness and tensile strength increased firstly and then decreased with the increasing V contents. The specimen 3# with 0.5 wt% V exhibits the excellent mechanical properties, with microhardness, tensile strength (Rm), yield strength (Re) and elongation of 500.1 HV, 1756 MPa, 1375 MPa, and 11.9% respectively, superior to the laser-cladded Fe-based MSS coatings reported in the literature.
(3) Better understanding corrosion resistance mechanism. A small amount of V element (0.25%) can improve the corrosion resistance of MSS coating. However, the corrosion resistance of the V-bearing coatings decreases successively with the increasing V contents over 0.25%. This is due to excessive precipitation of VN and *M*_23_C_6_, which partially consumed the element Cr.

This work will be greatly potential for facilitating laser manufacturing high-strength V-bearing steel coatings/components with excellent mechanical properties and corrosion resistance.

## Figures and Tables

**Table 1:** Chemical compositions (wt%) of the substrate and powders and the corresponding specimen denominations.

	Element	C	Cr	Ni	Si	Mn	V	Fe
Specimen								
Q235		0.18	-	-	0.26	0.55	-	Bal.
1# (0%V)		0.16	13.09	0.51	0.80	0.91	0	Bal.
2# (0.25%V)		0.16	13.09	0.51	0.80	0.91	0.25	Bal.
3# (0.5%V)		0.16	13.09	0.51	0.80	0.91	0.5	Bal.
4# (1%V)		0.16	13.09	0.51	0.80	0.91	1	Bal.

**Table 2:** Comparison of tensile mechanical properties of different laser-cladded MSS coatings.

Coatings	Carbon Content/wt%	R_m_/MPa	R_e_/MPa	Elongation/%	Treatment States
1# (0%V) MSS	0.16	1373 ± 23	1106 ± 38	5.9 ± 0.3	as-cladded
2# (0.25%V) MSS	0.16	1618 ± 14	1195 ± 52	10.1 ± 0.7	as-cladded
3# (0.5%V) MSS	0.16	1756 ± 35	1375 ± 83	11.9 ± 0.5	as-cladded
4# (1%V) MSS	0.16	1557 ± 49	1143 ± 60	13.2 ± 0.4	as-cladded
MSS [8]	0.01	1072	918	6.3	as-cladded
MSS [21]	0.19	1990	1750	7.3	as-cladded
420 MSS [25]	0.26	1802	1306	6.9	as-cladded
420 MSS [25]	0.26	1690	1109	15.8	as-tempered
431 MSS [29]	0.15	1283 ± 16	-	14.5 ± 1.5	as-heat treated
420 MSS [30]	0.35	1903 ± 34	1518 ± 75	2.7 ± 0.4	as-cladded
420 MSS [31]	0.23	1670 ± 202	-	13.9 ± 2.9	as-cladded

**Table 3:** Electrochemical corrosion parameters of the laser cladding layers with different V contents.

	Electrochemical	Corrosion Potential (SCE) E_corr_/V	Corrosion Current Density I_corr_/(A·cm^−2^)	Corrosion Rate /(mmpy)
Specimen				
1# (0%V)		−0.36375	7.0021 × 10^−6^	0.08044
2# (0.25%V)		−0.17458	3.4652 × 10^−6^	0.03369
3# (0.5%V)		−0.22494	2.2478 × 10^−4^	2.1859
4# (1%V)		−0.20589	2.8077 × 10^−4^	2.7302

## References

[1] Wu F., Chen T., Wang H.J., Liu D.F. (2017). Effect of Mo on Microstructures and Wear Properties of In Situ Synthesized Ti(C,N)/Ni-Based Composite Coatings by Laser Cladding. Materials.

[2] Hou X.C., Du D., Chang B.H., Ma N.S. (2019). Influence of Scanning Speed on Microstructure and Properties of Laser Cladded Fe-Based Amorphous Coatings. Materials.

[3] Zhang Z., Farahmand P., Kovacevic R. (2016). Laser cladding of 420 stainless steel with molybdenum on mild steel A36 by a high power direct diode laser. Mater. Des..

[4] Sun S.D., Fabijanic D., Ghaderi A., Learyl M., Toton J., Sun S.J., Brandt M., Easton M. (2016). Microstructure and hardness characterisation of laser coatings produced with a mixture of AISI 420 stainless steel and Fe-C-Cr-Nb-B-Mo steel alloy powders. Surf. Coat. Technol..

[5] Wang K.M., Chang B.H., Chen J.S., Fu H.G., Lin Y.H., Lei Y.P. (2017). Effect of molybdenum on the microstructures and properties of stainless steel coatings by laser cladding. Appl. Sci..

[6] Nath S.D., Clinning E., Gupta G., Poirier V.W., L’Espérance G., Gulsoy O., Kearns M., Atre S.V. (2019). Effects of Nb and Mo on the Microstructure and Properties of 420 Stainless Steel Processed by Laser-Powder Bed Fusion. Addit. Manuf..

[7] Zhang Z., Yu T., Kovacevic R. (2017). Erosion and corrosion resistance of laser cladded AISI 420 stainless steel reinforced with VC. Appl. Surf. Sci..

[8] Wen P., Cai Z.P., Feng Z.H., Wang G. (2015). Microstructure and mechanical properties of hot wire laser clad layers for repairing precipitation hardening martensitic stainless steel. Opt. Laser Technol..

[9] Medina S.F., Go´mez M., Rancel L. (2008). Grain refinement by intragranular nucleation of ferrite in a high nitrogen content vanadium microalloyed steel. Scripta Mater..

[10] Yang G.W., Sun X.J., Li Z.D., Li X.X., Yong Q.L. (2013). Effects of vanadium on the microstructure and mechanical properties of a high strength low alloy martensite steel. Mater. Des..

[11] Gwon H., Kim J.K., Shin S., Cho L., Cooman B. (2017). The effect of vanadium micro-alloying on the microstructure and the tensile behavior of TWIP steel. Mater. Sci. Eng. A.

[12] Li L.L., Song C.H., Lu J., Hu J.L., Zhou T. (2016). Precipitation Behavior and Microstructural Evolution of Vanadium-Added TRIP-Assisted Annealed Martensitic Steel. Steesl Res..

[13] David S.A., Vitek J.M., Reed R.W., Hebble T.L. (1987). Effect of Rapid Solidification on Stainless Steel Weld Metal Microstructures and Its Implications on the Schaeffler Diagram.

[14] Dong Y., Zhou K.Y., Lu Y.P., Gao X.X., Wang T.G., Li T.J. (2014). Effect of vanadium addition on the microstructure and properties of AlCoCrFeNi high entropy alloy. Mater. Des..

[15] Wang F.J., Zhang Y., Chen G.L. (2009). Atomic packing efficiency and phase transition in a high entropy alloy. J. Alloys Compd..

[16] Gulyaev A.P., Anashenko V.N., Karchevskaya N.I., Larina O.D., Matrosov Y.I. (1974). Solubility of vanadium and niobium nitrides in iron. Met Sci. Heat. Treat..

[17] Hui W.J., Chen S.L., Zhang Y.J., Shao C.W., Dong H. (2015). Effect of vanadium on the high-cycle fatigue fracture properties of medium-carbon microalloyed steel for fracture splitting connecting rod. Mater. Des..

[18] Dong J., Zhou X.S., Liu Y.C., Li C., Liu C.X., Guo Q.Y. (2017). Carbide precipitation in Nb-V-Ti microalloyed ultra-high strength steel during tempering. Mater. Sci. Eng. A.

[19] Shao Y., Liu C.X., Yan Z.S., Li H.J., Liu Y.C. (2018). Formation mechanism and control methods of acicular ferrite in HSLA steels: A review. J. Mater. Sci. Technol..

[20] Hua G.M., Li C.S., Cheng X.N., Zhao X.L., Feng Q., Li Z.J., Li D.Y., Szpunar J.A. (2018). First-principles study on influence of molybdenum on acicular ferrite formation on TiC particles in microalloyed steels. Solid State Commun..

[21] Li B.C., Zhu H.M., Qiu C.J., Gong X.K. (2020). Laser cladding and in-situ nitriding of martensitic stainless steel coating with striking performance. Mater. Lett..

[22] Cong Z.H., Jia N., Sun X., Ren Y., Almer J., Wang Y.D. (2009). Stress and Strain Partitioning of Ferrite and Martensite during Deformation. Metall. Mater. Trans. A.

[23] Lv Z., Xiao S., Xiao Z., Qian L., Wang W., Zhou Y., Fu W. (2017). Structural properties and bonding characteristic of interfaces between VN and VC from density functional calculations. J. Alloys Compd..

[24] Luo F., Yao J.H., Hu X.X., Chai G.Z. (2011). Effect of Laser Power on the Cladding Temperature Field and the Heat Affected Zone. J. Iron. Steel Res. Int..

[25] Zhu H.M., Li Y.Z., Li B.C., Zhang Z.Y., Qiu C.J. (2018). Effects of low-temperature tempering on micro-structure and properties of the laser-cladded AISI 420 martensitic stainless steel laser cladding layer. Coatings.

[26] Kumar V., Dixit U.S. (2018). A model for the estimation of hardness of laser bent strips. Opt. laser Technol..

[27] Chandra K., Kain V., Bhutani V., Raja V.S., Tewari R., Dey G.K., Chakravartty J.K. (2012). Low temperature thermal aging of austenitic stainless steel welds: Kinetics and effects on mechanical properties. Mater. Sci. Eng. A.

[28] Abbasi S.M., Shokuhfar A. (2007). Improvement of Mechanical Properties of Cr-Ni-Mo-Cu-Ti Stainless Steel with Addition of Vanadium. J. Iron. Steel Res. Int..

[29] Liu Y., Li A., Cheng X., Zhang S., Wang H. (2016). Effects of heat treatment on microstructure and tensile properties of laser melting deposited AISI 431 martensitic stainless steel. Mater. Sci. Eng. A.

[30] Sun S.D., Fabijanic D., Barr C., Liu Q.C., Walker K., Matthews N., Orchowski N., Easton M., Brandt M. (2018). In-situ quench and tempering for microstructure control and enhanced mechanical properties of laser cladded AISI 420 stainless steel powder on 300M steel substrates. Surf. Coat. Technol..

[31] Mohammad K.A., Mehdi M., Urbanic R.J., Edrisy A. (2020). Mechanical Behavior of Additive Manufactured AISI 420 Martensitic Stainless Steel. Mater. Sci. Eng., A.

[32] Park K., Nishiyama M., Nakada N., Tsuchiyama T., Takaki S. (2014). Effect of the martensite distribution on the strain hardening and ductile fracture behaviors in dual-phase steel. Mater. Sci. Eng. A.

[33] Bonagani S.K., Bathula V., Kain V. (2018). Influence of tempering treatment on microstructure and pitting corrosion of 13wt.% Cr martensitic stainless steel. Corros. Sci..

[34] Amezhnov A.V., Rodionova I.G., Batsalev A.I., D’yakonov D.L., Shaposhnikov N.G., Shatskii T.E., Marzoeva M.E. (2019). Effect of Chemical Composition and Microstructure Parameters on Carbon and Low-Alloy Steel Corrosion Resistance Under Oil Industry Pipeline Operation Conditions. Metallurgist.

[35] Qi X., Mao H.H., Yang Y.T. (2017). Corrosion behavior of nitrogen alloyed martensitic stainless steel in chloride containing solutions. Corros. Sci..

